# Phenotypes of severe asthma among children and adolescents in Brazil: a prospective study

**DOI:** 10.1186/s12890-015-0029-8

**Published:** 2015-04-17

**Authors:** Wenderson Clay Correia de Andrade, Laura Maria de Lima Belizário Facury Lasmar, Cristiane de Abreu Tonelli Ricci, Paulo Augusto Moreira Camargos, Álvaro A Cruz

**Affiliations:** Graduate Program in Health Sciences, Federal University of Minas Gerais, Belo Horizonte, Brazil; Pediatric Pulmonology Unit, University Hospital, Federal University of Minas Gerais, Avenida Alfredo Balena, 190, sala 267, Belo Horizonte, MG Brazil; ProAR-Center of Excellence in Asthma, Federal University of Bahia School of Medicine, Salvador, Brazil

**Keywords:** Refractory severe asthma, Difficult-to-treat asthma, Inflammatory biomarkers, Fractional exhaled nitric oxide, Sputum

## Abstract

**Background:**

The morbidity associated with severe uncontrolled asthma is disproportionately higher in low- and middle-income countries than in high-income countries. The aim of this study was to describe the phenotypic characteristics of difficult-to-treat severe asthma and treatment-resistant severe asthma in a sample of children and adolescents in Brazil.

**Methods:**

This was a prospective study, conducted between 2010 and 2014, following 61 patients (6–18 years of age) who had been diagnosed with severe uncontrolled asthma. The patients were classified and managed in accordance with the World Health Organization asthma follow-up protocol, which calls for re-evaluations of the diagnosis, level of control (functional and clinical), comorbidities, inhaler technique, and environmental factors, together with adjustment of the treatment to achieve a target level of control. We assessed pulmonary function, measured fractional exhaled nitric oxide, and performed sputum cytology. After the target rate of ≥ 80% adherence to inhaled corticosteroid treatment had been reached and all of the re-evaluations had been performed, the patients incorrectly diagnosed with severe uncontrolled asthma were excluded and the remaining patients were classified as having treatment-resistant or difficult-to-treat severe asthma.

**Results:**

We found that, of the 61 patients evaluated, 10 had been misdiagnosed (i.e., they did not have asthma), 15 had moderate asthma, and 36 had severe uncontrolled asthma. Among those 36 patients, the asthma was classified as treatment-resistant in 20 (55.6%) and as difficult-to-treat in 16 (44.4%). In comparison with the patients with difficult-to-treat severe asthma, those with treatment-resistant severe asthma showed a higher median level of fractional exhaled nitric oxide (40 ppb vs. 12 ppb; *P* < 0.037) and a lower median forced expiratory volume in one second (61% vs. 87%; *P* < 0.001).

**Conclusions:**

Although patients with treatment-resistant severe asthma cannot always be distinguished from those with difficult-to-treat severe asthma on the basis of baseline clinical characteristics, reduced airflow and elevated fractional exhaled nitric oxide are factors that could distinguish the two groups. Patients diagnosed with severe uncontrolled asthma should be re-evaluated on a regular basis, in order to exclude other diagnoses, to reduce exacerbations, and to identify patients with persistent airflow limitation.

## Background

Severe uncontrolled asthma comprises three categories [1]: untreated severe asthma; difficult-to-treat severe asthma; and treatment-resistant severe asthma. Those categories differ in terms of their connotation in the context of public health, as well as in terms of the challenges they pose. Failure to achieve and maintain asthma control can increase the risk and frequency of exacerbations, as well as increasing the risk of asthma-related death. Individuals with uncontrolled asthma are also more likely to have adverse reactions to asthma medications and to develop chronic morbidity, such as impaired pulmonary function. In children with uncontrolled asthma, lung growth can also be impaired [1].

Patients with severe uncontrolled asthma should be evaluated carefully. The evaluation of such patients should include the differential diagnosis of their symptoms, the determination of their access to medications, and the identification of potentially reversible risk factors (comorbidities, inefficient inhaler technique, poor adherence to the treatment regimen, and asthma triggers in the environment). In addition, attempts should be made to determine whether the use of maintenance medications is optimal [1-3]. The objective of such evaluations is to distinguish patients with treatment-resistant severe asthma, in whom the symptoms remain uncontrolled despite the highest level of recommended treatment or control can be maintained only with the highest level of recommended treatment, from those with difficult-to-treat severe asthma, in whom control can be achieved after the removal of reversible risk factors [1]. It is important to make the distinction between treatment-resistant severe asthma and difficult-to-treat severe asthma, in order to avoid unnecessary invasive procedures, minimize adverse effects of asthma medications, and hold down health care costs, given that asthma control is possible in patients with difficult-to-treat severe asthma, whereas those with treatment-resistant severe asthma are candidates for clinical trials of other treatment strategies [1,3].

There have been few studies aimed at distinguishing between difficult-to-treat severe asthma and treatment-resistant severe asthma in children and adolescents. Studies conducted in high-income countries have reported inconsistent findings [4,5]. Some authors have suggested that patients with difficult-to-treat severe asthma show better baseline pulmonary function and lower fractional exhaled nitric oxide (FeNO) than do those with treatment-resistant severe asthma [4]. In contrast, others have found that neither FeNO nor forced expiratory volume in one second (FEV_1_) discriminate between those two phenotypes of severe uncontrolled asthma [5].

According to data from the International Study of Asthma and Allergies in Childhood [6], the prevalence of asthma among 13- to 14-year-olds in Brazil is approximately 20%. The morbidity associated with severe uncontrolled asthma is disproportionately higher in low- and middle-income countries (LMICs) than in high-income countries [1,7], which calls attention to the need for studies aimed at identifying strategies for reducing asthma-related morbidity in LMICs. A group of experts assembled by the World Health Organization (WHO) has proposed a protocol for severe asthma follow-up and intervention, which defines three categories of severe uncontrolled asthma [1]. The objective of the present study was to describe the phenotypes of severe uncontrolled asthma in a sample of children and adolescents in Brazil.

## Methods

This was a prospective study conducted between June 2010 and May 2014 at a referral center for severe asthma, the Multidisciplinary Center for Difficult-to-Control Asthma of the Federal University of Minas Gerais *Hospital das Clínicas* located in the city of Belo Horizonte, in southeastern Brazil. The center, which operates under the auspices of the Belo Horizonte Municipal “Wheezy Child” Program [8], serves an area in which most families are of low socioeconomic status, providing them with asthma medications and spacers for inhalers, all at no cost to the families. Children and adolescents with asthma are referred from primary care physicians to pediatric pulmonologists, who in turn refer them to our facility. Consequently, the patients evaluated in this study all suffered from asthma that could not be controlled in primary or secondary care. We defined a diagnosis of severe uncontrolled asthma as described by the WHO [1]: “Uncontrolled asthma which can result in risk of frequent severe exacerbations (or death) and/or adverse reactions to medications and/or chronic morbidity (including impaired lung function or reduced lung growth in children)”.

Severe exacerbations were defined as those that required the use of systemic corticosteroids, unscheduled visits to the emergency room, or emergency hospital admissions [1].

- The categories, or phenotypes, of severe uncontrolled asthma were defined as follows [1-3]: untreated severe asthma—asthma for which the treatment is not appropriate to the degree of severity
- difficult-to-treat severe asthma—asthma in which a lack of control or partial control is attributed to other factors (lack of access to asthma medications, poor adherence to treatment, incorrect inhaler technique, exposure to tobacco smoke, exposure to allergens in the environment, and psychosocial stressors)
- treatment-resistant severe asthma—asthma that is uncontrolled or only partially controlled despite the use of high doses of an inhaled corticosteroid (ICS) in combination with a long-acting β_2_ agonist (LABA), with or without the regular or constant use of systemic corticosteroids, or asthma that can be controlled only with such aggressive treatment

### Patients

We included all 6- to 18-year-old patients referred to our facility with a suspected diagnosis of severe uncontrolled asthma. Those patients were re-evaluated once every three months at our facility.

### Assessments

The patients were evaluated in accordance with the severe asthma follow-up protocol proposed by the WHO consultants, which is designed to characterize the phenotypes of severe uncontrolled asthma [1]. To distinguish between treatment-resistant severe asthma and difficult-to-treat severe asthma [1,2], the WHO protocol includes the following: re-evaluation of the level of asthma control; re-evaluation of the diagnosis; re-evaluation of the factors associated with a lack of control (comorbidities, lack of treatment adherence, incorrect inhaler technique, and environmental factors); and adjustment of the treatment to a level appropriate to the level of control.

#### Evaluation of the level of asthma control

We evaluated the level of asthma control in accordance with the criteria established by the Global Initiative for Asthma [9], utilizing the following parameters: daytime symptoms; nighttime symptoms; ability to perform physical activities; occurrence of exacerbations; the need for rescue medication; and the FEV_1_. We also applied the Asthma Control Test (ACT), on which a score < 20 (out of a maximum of 25) indicates a lack of control [10].

Functional control of asthma was evaluated by spirometry (Spirobank® II; MIR, Rome, Italy), in accordance with the recommendations of the American Thoracic Society [11]. Before and after the administration of a bronchodilator (400 μg of albuterol) by pressurized metered-dose inhaler (pMDI), we measured FEV_1_, forced vital capacity (FVC), the FEV_1_/FVC ratio, and forced expiratory flow between 25% and 75% of the vital capacity (FEF_25–75%_). A post-bronchodilator increase in FEV_1_ of 200 ml or ≥ 12% was considered significant [12]. All parameters are expressed as a percentage of the predicted value, based on age, gender, and height [12,13].

#### Confirmation of the diagnosis of the asthma

Patients in whom asthma control was not achieved after their comorbidities had been evaluated and their treatment regimen had been adjusted were submitted to clinical assessment in order to rule out alternative diagnoses [1-3]. When clinically indicated (in 66.7% of the patients), we performed high-resolution computed tomography scans of the chest, with slices during inspiration and expiration. We also determined chloride concentrations in sweat (in all of the patients); monitored esophageal pH (in 38.9%); administered tuberculin skin tests (in all of the patients); conducted immunological testing, determining immunoglobulin levels (in all of the patients), immunophenotyping lymphocytes (in 11.1%), and quantifying the response to vaccine antigens (in all of the patients); and performed bronchoscopy (in 8.3%). In one case, we studied the morphology of the cilia under electron microscopy. When indicated, we also performed cardiological, otorhinolaryngological, and psychiatric evaluations (in 44.4%, 38.9%, and 8.3% of the patients, respectively) [1-3].

#### Evaluation of associated factors

##### Allergic rhinitis and allergic sensitization

Diagnoses of persistent allergic rhinitis were re-evaluated in accordance with the criteria of the Allergic Rhinitis and its Impact on Asthma initiative [14], which classify allergic rhinitis as persistent or intermittent, as well as grading it as mild, moderate, or severe. Those determinations are made on the basis of one or more of the six signs and symptoms of allergic rhinitis (oropharyngeal pruritus, sneezing, watery rhinorrhea, nasal pruritus, ocular pruritus, and nasal obstruction) being present for four or more days in a week and for more than four consecutive weeks [14]. We also employed an adapted clinical scale for rhinitis [15], which has previously been used in Brazil [16]. Each of the six parameters (nasal pruritus, oropharyngeal pruritus, ocular pruritus, rhinorrhea, sneezing, and nasal obstruction) is scored from 0 (better) to 3 (worse), the maximum possible score therefore being 18.

Allergic sensitization was defined as positivity on a skin prick test (ALK-Abelló, Hørsholm, Denmark). Test results were considered positive when the papule size was 3 mm greater than was that of the negative control. The following allergens were tested: *Dermatophagoides pteronyssinus, Dermatophagoides farinae, Blomia tropicalis, Alternaria alternata*, *Aspergillus fumigatus,* cat dander, dog dander, and cockroach allergens (from *Periplaneta americana* and *Blattella germanica*). The positive and negative controls were histamine and saline solution, respectively [17].

We determined serum levels of total immunoglobulin E with a fluorescence enzyme immunoassay (ImmunoCAP; Phadia, Uppsala, Sweden), using the reference values based on age range [18].

##### Inhaler technique

Patient inhaler technique was evaluated on the basis of the recommendations for the proper use of pMDIs and dry powder inhalers (DPIs) and was reviewed at every visit. The pMDIs were used with a spacer attached to the mouthpiece [19].

##### Psychosocial and emotional factors

Patients with anxiety or depressive disorders were identified as outlined in the Global Initiative for Asthma [9]. Those so identified were evaluated and treated by a psychiatrist.

##### Gastroesophageal reflux disease

Patients with gastroesophageal reflux disease, as evidenced by epigastric pain or heartburn, underwent 24-h pH monitoring and, if necessary, upper gastrointestinal endoscopy [10].

##### Adherence to treatment

The rate of adherence to treatment with an ICS was obtained by calculating what percentage of the total number of recommended doses during a given period were taken by the patient. To that end, we consulted the dose counters of the inhalers, counted the empty DPI capsules, and checked those counts against the records of the dates on which the medications were dispensed [1,8,20].

### Evaluation of the environment

We classified the home environment as uncontrolled when the parents or guardians reported the presence of mold, dust mites, pets, or smoking in the home [2,9]. Measures aimed at reducing exposure (using washable covers on mattresses, dusting furniture with a damp cloth, keeping pets outside, and prohibiting smoking within the home) were recommended as necessary [21].

### Evaluation of sputum cellularity

We induced sputum with an ultrasonic nebulizer (Devilbiss Healthcare, Langen, Germany), in accordance with the recommendations of the European Respiratory Society [22]. Patients with clinical signs and symptoms of infection were exempted from sputum induction. In patients with stable asthma who showed post-bronchodilator values of FEV_1_ ≥ 60% of predicted, we used 4.5% hypertonic saline, whereas we used normal saline in those who showed post-bronchodilator values of FEV_1_ < 60%. The processing of the induced sputum samples was also performed in accordance with the recommendations of the European Respiratory Society. Samples were considered usable if they contained ≤ 20% squamous cells, showed ≥ 50% cell viability, and had ≥ 400 inflammatory cells [22,23]. The pattern of cellularity was defined as follows [22,23]: eosinophilic (when eosinophils accounted for ≥ 2.5% and neutrophils accounted for ≤ 54%); neutrophilic (when eosinophils accounted for < 2.5% and neutrophils accounted for > 54%); or paucicellular (when eosinophils accounted for < 2.5% and neutrophils accounted for < 54%).

### Measurement of FeNO

In all cases, FeNO was measured prior to the spirometry and when the patient was free of upper airway infections. Using a portable analyzer (NIOX MINO; Aerocrine AB, Solna, Sweden), we obtained the FeNO values at an expiratory flow rate of 50 ml/s [24].

### Optimizing the treatment

We optimized the treatment in accordance with the level of control [7]. The patients were using DPIs delivering a combination of budesonide and formoterol, either Symbicort (AstraZeneca, Lund, Sweden) or Alenia (Aché Laboratórios Farmacêuticos S.A., Guarulhos, Brazil); or DPIs or pMDIs containing fluticasone combined with salmeterol (Seretide; GlaxoSmithKline, Stevenage, UK), montelucaste (Montelair; Aché Laboratórios), oral prednisolone (generic), or omalizumab (Xolair; Novartis Biociências S.A., São Paulo, Brazil).

### Statistical analysis

Before and after the interventions recommended by the WHO consultants, we performed comparative analyses using McNemar’s test and the Wilcoxon test. In our analysis, we included only those patients who were ≥ 80% adherent to the treatment regimen, dividing them into two groups on the basis of whether or not clinical and functional control of the asthma was achieved [1-3]: difficult-to-treat severe asthma (in which such control was achieved); and treatment-resistant severe asthma (in which it was not). We then evaluated the groups in terms of the baseline characteristics that might differentiate between the two, using the chi-square test (with Yates’ correction or Fisher’s exact test), or the Mann–Whitney test, as necessary. The level of statistical significance was set at 5%.

### Ethical aspects

The study was approved by the Federal University of Minas Gerais Research Ethics Committee (Protocol no. 149/10). All participating patients or their legal guardians gave written informed consent.

## Results

We found that 10 (16.4%) of the 61 patients evaluated had been misdiagnosed (i.e., they did not have asthma), 15 (24.6%) had moderate asthma, and 36 (59.0%) had severe uncontrolled asthma. The alternative diagnoses included bronchiolitis obliterans following an infection (in six patients), cystic fibrosis (in one), primary ciliary dyskinesia (in one), pulmonary tuberculosis (in one), and a sequela of bronchopulmonary dysplasia (in one). Those 36 patients comprised our study sample. Table 1 shows the baseline demographic, anthropometric, and clinical characteristics of the patients.

**Table 1:** **Baseline characteristics of pediatric patients with severe uncontrolled asthma (N = 36) treated at a referral center for asthma in Brazil**

**Variable**	**n (%)**	**Median (IQR)**
Female gender	23 (63.9)	
Age (years)		11.6 (6.2 to 17.8)
Height (Z score)		0.05 (−2.66 to 1.00)
BMI (Z score)		−0.48 (−2.90 to 1.84)
Well-controlled home environment	9 (25.0)	
Severe persistent allergic rhinitis	35 (97.2)	
Severe exacerbations in the last 12 months		5 (1 to 20)
Allergic rhinitis score		8 (0 to 18)
Gastroesophageal reflux disease	7 (19.4)	
Psychiatric disorder	3 (8.3)	
Smoking in the home (reported by the residents)	12 (33.3)	
Fully adherent to treatment	2 (5.6)	
Previous pulmonary function testing	16 (44.4)	
Incorrect inhaler technique	13 (36)	
Previous ICU admission	7 (19.5)	
Uncontrolled asthma (GINA criteria)	36 (100)	
Posology and medications in use		
Equivalent dose of budesonide (μg)		800 (800 to 1200)
ICS alone	9 (25.0)	
ICS + LABA	27 (75.0)	
Leukotriene receptor antagonists	4 (11.1)	
Continuous oral ICS	3 (8.3)	
Omalizumab	0 (0.0)	

IQR: interquartile range; BMI: body mass index; ICU: intensive care unit; GINA: Global Initiative for Asthma.

In the sample as a whole, asthma-related morbidity was high, with a median of five exacerbations in the last 12 months. All of the patients had uncontrolled asthma and were taking high doses of ICS, which is characteristic of severe uncontrolled asthma. Despite the lack of control, 25% of the patients were being treated with regimens that did not include a LABA and were therefore classified as receiving treatment that was not appropriate to the level of control. The majority of the patients had severe persistent allergic rhinitis and were exposed to dust mite allergens and tobacco smoke in their environment. In addition, 36% used incorrect inhaler techniques, the most common errors being not exhaling completely before inhaling the medication and not inhaling deeply enough. Table 2 shows the characteristics of the patients before and after the interventions recommended by the WHO consultants [1]. After the interventions, there was an increase in the proportion of patients showing correct inhaler technique, together with better rhinitis scores, better ACT scores, and an increase in the use of leukotriene receptor antagonists. In addition, there were post-intervention reductions in the number of exacerbations, in the FeNO levels, and in the post-bronchodilator variations in FEV_1_ and FEF_25–75%_, as well as an increase in the absolute values for FEV_1_.

**Table 2:** **Characteristics of pediatric patients with severe uncontrolled asthma (N = 36) before and after application of the WHO protocol** ^**a,b**^

**Variable**	**Pre-protocol**	**Post-protocol**	***P***
Correct inhaler technique	23 (63.9)	31 (86.1)	0.008
Fully adherent to treatment	2 (5.6)	34 (94.4)	N/A
Rhinitis score	8 (0–18)	5 (0–15)	0.001
Asthma Control Test score	16.5 (6–25)	22.5 (8–25)	0.002
Posology and medications in use			
Leukotriene receptor antagonists	4 (11.1)	12 (33.3)	0.008
Omalizumab	0 (0)	1 (2.7)	N/A
Continuous oral ICS	3 (8.3)	8 (22.2)	0.063
Equivalent dose of budesonide (μg)	800 (800–1200)	800 (800–1600)	0.059
Well-controlled home environment	9 (25)	15 (41.7)	0.109
Psychiatric disorder	3 (8.3)	8 (22.2)	0.125
Number of exacerbations	4 (0–20)	2 (0–11)	0.006
Sputum cytology^**c**^			
Eosinophils (%)	2.0 (0–37)	1.1 (0–49)	0.948
Eosinophils ≥ 2.5%	9 (25.0)	9 (25.0)	0.362
Neutrophils > 54%	4 (17.4)	6 (18.8)	0.114
Pre-BD FEV_1_ (%)	77.0 (36–113)	78.7 (31–113)	0.038
Pre-BD FEV_1_/FVC ratio (%)	78.0 (53–100)	80.0 (43–95)	0.417
Pre-BD FE _25–75%_ (%)	61.0 (16–131)	66.5 (15–110)	0.156
Post-BD variation in FEV_1_ (%)	11.0 (0–70)	5.0 (0–45)	0.004
Post-BD variation in FEF_25–75%_ (%)	31.0 (0.0–166.0)	14.0 (0.0–96.0)	0.013
FeNO (ppb)	31 (5–125)	14 (0–112)	0.016

^**a**^Values expressed as n (%) or as median (range); ^**b**^median time between the pre- and post-protocol evaluations: 6.07 months (range, 0.7–24.27 months); ^**c**^some patients were unable to produce usable sputum samples: 13 in the pre-protocol evaluation; and 4 in the post-protocol evaluation. BD: bronchodilator.

Table 3 shows the comparative analysis between the patients with treatment-resistant severe asthma and those with difficult-to-treat severe asthma. At baseline, FEV_1_ was 61% and 87% of the predicted value in the patients with treatment-resistant severe asthma and difficult-to-treat severe asthma, respectively, and the pre-bronchodilator values of FEV_1_, FEV_1_/FVC ratio, and FEF_25–75%_ were lower in the patients with treatment-resistant severe asthma than in those with difficult-to-treat severe asthma, as were the post-bronchodilator variations in FEV_1_ and FEF_25–75%_. After the interventions, 20 (55.6%) of the 36 patients were classified as having treatment-resistant severe asthma and 16 (44.4%) were classified as having difficult-to-treat severe asthma. We found that the two phenotypes were indistinguishable on the basis of baseline clinical characteristics or atopy, although a distinction between the two was suggested on the basis of the baseline spirometry results and level of FeNO. In the sample as a whole, the median time to achieving ≥ 80% adherence to treatment was six months, and there was no statistical difference between the two groups in terms of that parameter.

**Table 3:** **Baseline characteristics of pediatric patients with severe uncontrolled asthma (N = 36) treated at a referral center for asthma in Brazil, by phenotype** ^**a**^

**Variable**	**Severe uncontrolled asthma**		***P***
	**Treatment-resistant asthma**	**Difficult-to-treat asthma**	
	**(n = 20)**	**(n = 16)**	
Female gender	13 (65.0)	10 (62.5)	0.846
Age (years)	12.0 (6.4–17.8)	11.0 (6.2–17.7)	0.514
Pattern of sputum cellularity^**b**^			
Eosinophilic	5 (31.3)	1 (14.3)	0.52
Neutrophilic	3 (18.8)	1 (14.3)	
Paucicellular^**a**^	6 (37.5)	4 (57.1)	
Mixed	2 (12.5)	1 (14.3)	
Number of ICU admissions	0.0 (0.0–6.0)	0.0 (0.0–4.0)	0.621
Courses of oral corticosteroids in the last 12 months (n)	0.5 (0.0–7.0)	1.00 (0.0–12.0)	0.143
Exacerbations in the last 12 months	6 (1–12.0)	4 (1–20.0)	0.311
Time to achieve ≥ 80% adherence and complete the protocol (months)	6.2 (0.7–24.2)	5.8 (1.4–17.3)	0.987
Asthma Control Test score	14 (6–24)	18 (10–24)	0.091
Rhinitis score	7.5 (4–15)	9 (0–15)	0.886
FVC (% of predicted)	74.9 (59.6–106.7)	93.6 (81.3–117)	<0.001
FEV_1_ (% of predicted)	61.0 (36.0–98.6)	87.0 (72.1–113)	<0.001
FEV_1_/FVC ratio	78.3 (43.1–86.1)	84.3 (72.1–98.9)	0.009
FEF_25–75%_ (%of predicted)	39.8 (16.0–83.1)	74.6 (29.1–131.3)	0.002
Post-BD variation in FEV_1_ (%)	17.0 (1.7–70.0)	6.2 (0.0–38.3)	0.012
Post-BD variation in FEF_25–75%_ (%)	38.9 (1.4–166.0)	24.6 (0.0–59.2)	0.031
Equivalent dose of budesonide (μg)	800 (800–1200)	800 (800–800)	0.975
FeNO (ppb)	40 (7–125)	12 (5–53)	0.037
Total IgE total (mg/dl)	1040 (65–3000)	454 (11–2500)	0.098
Sensitization to dust mite allergens	20 (100)	12 (80)	0.069
Sensitization to fungi	7 (35)	5 (33.3)	0.603
Sensitization to pet dander	5 (25)	4 (26.7)	0.605
Sensitization to cockroach allergens	4 (20)	3 (20)	0.659

^**a**^Values expressed as n (%) or as median (range); ^**b**^some patients were unable to produce usable sputum samples: 4 in the treatment-resistant group; and 9 in the difficult-to-treat group. ICU: intensive care unit; BD: bronchodilator; IgE: immunoglobulin E.

## Discussion

We found that 16.4% of the patients referred with a diagnosis of severe uncontrolled asthma did not have asthma at all. This finding is in agreement with those of other studies, in which 12–50% of such patients were found to have been misdiagnosed [25]. This underscores the importance of re-evaluating the diagnosis of asthma in patients who do not achieve control [1,9].

At baseline, the majority of our patients were under treatment with high doses of ICS and LABA, which is a reflection of the fact that those medications are made readily available by the health care system in the area served by our referral center. A consensus statement recently published jointly by the European Respiratory Society and the American Thoracic Society provided age-specific guidelines for the appropriate doses of inhaled corticosteroid in cases of “severe therapy-resistant asthma” [26]. The median age of our patients was 11.6 years (range, 6.2–17.8 years). our patients who were ≤ 12 years of age were treated with a daily dose of 800 μg of inhaled budesonide or equivalent, which the aforementioned guidelines classify as a high dose for patients in this age group [26]. The patients who were referred from health care systems in which LABAs are not made available accounted for the fact that 25% of the patients were not receiving treatment appropriate to the severity of their asthma. In addition, other asthma medications, such as leukotriene receptor antagonists and omalizumab, were unavailable to those patients. It is important that all effective asthma medications are made available and accessible to asthma patients, which is not always the case in LMICs [1,2]. In Brazil, a study designed to estimate the direct and indirect costs of severe asthma, as well as the overall economic impact of asthma, conducted under the auspices of the Bahia State Program for the Control of Asthma and Allergic Rhinitis, showed that the costs of asthma treatment consume, on average, 24% of the total family income in the city of Salvador (capital of the state of Bahia) [27]. When the indirect costs were included, that figure rose to 29%. The participants in the present study had free access to appropriate medications via the public health care system.

It is of note that, at baseline, asthma was uncontrolled in all of the patients, even those for whom high doses of ICS and LABA had been prescribed. In the majority of cases, there was no documentation of the medication having been dispensed and the patients did not have their inhalers with them, which precluded the counting of the doses. Therefore, prescribing a given medication does not guarantee that it will be administered.

One of the major factors associated with a lack of asthma control is poor adherence to treatment. Despite the fact that there are no fully reliable methods of verifying such adherence, it is recommended that it be quantified with one of the methods available, given that the definition of severe uncontrolled asthma is based on of the dosage required to effectively achieve and maintain control of the symptoms [1,20]. Within the health care systems that provide asthma medications, dispensing records could play an important role by allowing patients who do not refill their prescriptions to be identified. Thus, pharmacists could be on the front lines of the battle to improve treatment adherence, which is particularly important in patients with severe asthma. Because poor adherence to treatment is one of the major determinants of severe uncontrolled asthma, we opted to evaluate the patients in our sample only after they had reached a rate of adherence ≥ 80%.

At baseline, it was not possible to determine the rate of adherence to treatment. In our sample, the median time required to reach ≥ 80% adherence was six months. We found it surprising that it took so long for our patients to achieve a satisfactory rate of adherence, especially because they were already receiving appropriate treatment, which made it impossible to increase the dosages or add other recommended medications during that period.

Sharples et al. reported that 41% of patients with treatment-resistant severe asthma were ≥ 80% adherent to treatment, compared with only 24% of those with difficult-to-treat severe asthma [4]. In the present study, we distinguished between the two phenotypes only after ≥ 80% adherence had been achieved. The authors of an earlier study of asthma treatment in children and adolescents concluded that the rate of adherence reported by parents or guardians is higher than is the true (measured) rate [28]. In a study of children with treatment-resistant severe asthma [5], another group of authors used the reported rates of adherence but questioned the reliability of those data, suggesting that some method of quantifying adherence should be employed, which would allow poorly adherent individuals to be identified.

Despite the prerequisite of a documented ≥ 80% rate of adherence, 55.6% of our patients did not achieve clinical or functional asthma control. This finding is consistent with those of Sharples et al., who reported that, among the patients with uncontrolled asthma, the rate of adherence was 60.3% in those with treatment-resistant severe asthma, compared with 39.7% in those with difficult-to-treat severe asthma [4].

By applying the interventions suggested by the WHO panel of experts, we were able to increase the rate of adherence, improve ACT scores, increase the proportion of patients using correct inhaler techniques, improve rhinitis scores, optimize the treatment regimens, and maximize the environmental control, which is considered essential. Although it is not always easy or simple to achieve, it is important that these essential measures are taken. In the present study, we found that the interventions applied resulted in a satisfactory level of environmental control, despite the fact that we were able to eliminate smoking from the home environment (as determined on the basis of self-reported smoking cessation on the part of the residents) in only six of the cases, in which family members who reported smoking in the home had been referred to the smoking cessation outpatient clinic of a university hospital for treatment. It is possible that the environment could have been controlled more efficiently had home visits occurred [21].

Differentiating between treatment-resistant severe asthma and difficult-to-treat severe asthma has important implications for clinical practice. Patients with treatment-resistant severe asthma cannot achieve a satisfactory level of control even when potential risk factors are removed, such patients being candidates for clinical trials of new treatment strategies. In patients with treatment-resistant severe asthma, evaluations that are more invasive can be justified [29]. In patients with difficult-to-treat severe asthma, however, control can be achieved when the essential measures are taken, resulting in fewer exacerbations, social reintegration of the patients, and a reduction in health care costs.

In the present study, it was not possible to distinguish between the patients with treatment-resistant severe asthma and those with difficult-to-treat severe asthma on the basis of their baseline clinical characteristics (including ACT and rhinitis scores) or clinical history (number of exacerbations and number of admissions to the intensive care unit). That might explain, in part, the high morbidity that both groups of patients exhibited at baseline. It is possible to distinguish between the two phenotypes only after the essential measures have been taken, which is difficult to achieve outside of a specialized treatment facility. However, we identified markers that are suggestive of a distinction between treatment-resistant and difficult-to-treat severe asthma prior to any intervention, such markers including spirometry values and the FeNO level.

We found statistically significant baseline differences between the patients with treatment-resistant severe asthma and those with difficult-to-treat severe asthma in terms of the pre-bronchodilator FEV_1_, FEV_1_/FVC ratio, and FEF_25–75%_, as well as the post-bronchodilator variations in FEV_1_ and FEF_25–75%_. Sharples et al. observed such a difference, although only for post-bronchodilator variations in FEV_1_, whereas Konradsen et al. found no difference in FEV_1_ values between the two phenotypes [5]. The latter group of authors reported that the parental level of education and family income were both lower among the patients with treatment-resistant severe asthma [5]. In the present study, all of the patients belonged to families with monthly incomes below US$ 600.00 and only one patient had a parent with more than a high school education.

In our sample, the median FeNO values at baseline were higher in the patients with treatment-resistant severe asthma than in those with difficult-to-treat severe asthma (40 ppb vs. 12 ppb). Sharples et al. also showed that FeNO discriminates between the two phenotypes [4], although Konradsen et al. found no such distinction [5]. Despite the statistical significance observed in the present study, the viability of monitoring FeNO in LMICs has to be questioned. Although the determination of FeNO is a non-invasive procedure that is easily performed, it is too costly to be incorporated into the routine evaluation of all patients suspected of having severe asthma.

In another study evaluating induced sputum samples collected from children and adolescents, Bossley et al. reported that the median proportion of eosinophils was 7.5% in the atopic patients with treatment-resistant severe asthma, among whom the median age was 11.8 years of age [30]. In a study comparing controlled severe asthma with mild asthma in children and adolescents, no significant differences were found between the two types in terms of the proportion of eosinophils in induced sputum [31]. In the present study, we also found no significant difference between treatment-resistant severe asthma and difficult-to-treat severe asthma in terms of the pattern of cellularity in sputum. Nevertheless, it is notable that sputum eosinophilia persisted in 25% of our patients even after the treatment regimen had been optimized.

In a study of sputum induction in children with difficult asthma, Lex et al. found that 11% had sputum neutrophilia [23]. The authors pointed out that, although all of the children evaluated were chronically symptomatic, only a minority had sputum neutrophilia or eosinophilia. One possible explanation proposed by the authors is that the high doses of corticosteroids used in treating those children might have altered the sputum cytology [23]. In the present study, we identified sputum neutrophilia in 18.8% of the treatment-resistant severe asthma group patients who were able to produce usable sputum samples in the post-protocol evaluation, none of whom had shown any clinical signs or symptoms of respiratory tract infection. It is possible that this was also a side effect of treatment, given that our patients were using high doses of inhaled corticosteroids, which have been shown to inhibit neutrophil apoptosis [32].

The measurement of pulmonary function variables is important to the evaluation of asthma control [1,9]. In our sample as a whole, 44.4% of the patients had never undergone pulmonary function testing. They presented with baseline values of 77% and 78% for FEV_1_ and the FEV_1_/FVC ratio, respectively, which are indicative of mild obstructive lung disease, as would be expected. In some studies, FEV_1_ has been reported to be only slightly altered in children with severe uncontrolled asthma [3]. In the present study, baseline FEV_1_ was comparable between the patients with treatment-resistant severe asthma and those with difficult-to-treat severe asthma. Therefore, it was possible to distinguish between the two phenotypes only by evaluating pulmonary function in detail. Even at the end of the follow-up period (after the WHO protocol had been applied and a high rate of adherence to treatment had been achieved), pulmonary function was still abnormal in 55.6% of our patients. That might be attributable to airway remodeling. It is possible that this population of patients could benefit from treatment with medications that have yet to be standardized for use in pediatric patients. It is also possible that, in LMICs, there is a large contingent of individuals with severe asthma featuring persistent airway limitation. Studies investigating this issue are warranted. There is also a need for studies evaluating the medium- and long-term risks of persistent airway limitation in children and adolescents in LMICs. A study conducted in high-income countries showed that children with severe asthma are at increased risk of developing adult COPD in adulthood, even if they never become smokers. The fixed abnormalities in lung function in adult life are established in early childhood and track at lower values progressing to persistent airway limitation in adulthood [33].

Evaluating 45 atopic pediatric patients with treatment-resistant severe asthma, Bossley et al. observed thickening of the basement membrane and of the smooth muscle layer, indicating airway remodeling [30]. In those same patients, the authors found that the mean baseline FEV_1_ was 66% of the predicted value, comparable to the 61% observed in our sample. The authors suggested that there is a real need for new therapeutic strategies targeting children and adolescents with asthma that cannot be controlled even with high doses of inhaled or oral corticosteroids [34], having also noted that the strategies employed in adult asthma patients cannot necessarily be extrapolated to children and adolescents [30]. Konradsen et al. reported mean baseline FEV_1_ values of 86% and 76% in their patients with treatment-resistant and difficult-to-treat severe asthma, respectively [5].

Some authors have identified a statistically significant difference between treatment-resistant and difficult-to-treat severe asthma in terms of allergic sensitization to aeroallergens, which was found to be more common in pediatric patients with the treatment-resistant form [4]. In the present study, we found no such distinction. In our sample as a whole, all but two of the patients tested positive for allergic sensitization. One of the patients in our treatment-resistant severe asthma group tested positive for sensitivity to *Aspergillus fumigatus*. Twelve patients (five in our difficult-to-treat severe asthma group and seven in our treatment-resistant severe asthma group) tested positive for sensitivity to *Alternaria alternata*, although computed scans of the chest revealed no bronchiectasis in any of those patients. Fungal sensitization has been associated with greater asthma severity [35].

One of the limitations of our study is that the sample was highly selected, given that it comprised patients seen at a referral center for severe asthma. However, the patients had been referred from secondary health care facilities and required an approach that involved asthma treatment at all levels of health care.

## Conclusions

Although patients seen at a referral center for severe asthma constitute a relatively homogeneous group, higher FeNO levels and greater impairment of pulmonary function at baseline are suggestive of treatment-resistant severe asthma. In our sample of children and adolescents with severe uncontrolled asthma in Brazil, we found that the evaluations and interventions proposed by the WHO consultants were effective. After the WHO protocol had been applied, we observed fewer exacerbations, better symptom scores, a higher proportion of patients using the correct inhaler technique, and higher rates of adherence to treatment. The use of the WHO protocol allowed us to exclude other diagnoses and to demonstrate that, despite appropriate treatment, many of the patients continued to show functional impairment.

To address the disproportionately high rates of morbidity associated with severe uncontrolled asthma in LMICs, we need to do more than simply increase the availability of asthma medications. In patients with severe uncontrolled asthma, we need to monitor adherence to treatment and the level of asthma control on a regular basis, which can best be achieved at a referral center with asthma specialists on staff. For certain patients, new treatment strategies need to be developed.

## Abbreviations

ACT: Asthma Control Test
DPI: Dry powder inhalers
FEF_25–75%_: Forced expiratory flow between 25% and 75% of the vital capacity
FeNO: Fractional exhaled nitric oxide
FEV_1_: Forced expiratory volume in one second
FVC: Forced vital capacity
ICS: Inhaled corticosteroid
LABA: Long-acting β_2_ agonist
LMICs: Low- and middle-income countries
pMDI: Pressurized metered-dose inhaler
post-BD: Post-bronchodilator
WHO: World Health Organization
